# Progress in Obstetrics

**Published:** 1896-06-13

**Authors:** 


### Progress in Obstetrics.

**Osteomalacia.**—This formidable though, fortunately, rare disease, presents difficulties and is of interest to every branch of our profession—to the obstetrician from the association of the complaint with pregnancy, and the dangers that may arise from it at childbirth; to the surgeon in his general care of the bony framework, and from the advocacy by some foreign operators, as remedial measures, of öophorectomy, or extirpation of the uterus; and to the physician from the obscure nature of the lesion, and the hopeless treatment given in the text-books.

Dr. Allison[1] records a case in which the diagnosis rests on a history of insidious onset, with weakness and lassitude in an individual previously well formed and healthy, followed by rheumatoid pains and softening of the bones of the lower extremities, which gave way below the knees, and afterwards failed to support the weight of the body, this being succeeded by softening of the bones of the trunk, and progressive contortion of the framework generally. The upper extremities became involved also, but the outlines of the bones of the head and face have not materially altered. The pelvis was characteristic in that the ischial tuberosities approximate; this has been allowed by partial dislocation backward of the sacrum. Phosphates in excess were in the urine. The patient was aged forty-three, was supposed to be healthy at birth, but was kept on the breast until fifteen months old; she was strong up to the age of thirteen, when the changes incident on puberty appear to have set up the present disease, which has run a progressive but unusually chronic course. Up to the age of twenty-seven, or fourteen years after, only the legs seemed to be affected, and it was not noticed until the age of thirty that the pelvis was abnormal. From thirty to forty-three years of age the spine, ribs, and both upper extremities have gradually become affected. This case has been treated by the administration of healthy bone-marrow, the preparation employed being a glycerine extract from the cancellous tissue of the leg bones of the calf (Brady and Martin). This was given in doses of half to one and a half drachms three times daily after food, the quantity used during the first three months being four ounces weekly.

Visible improvement was seen after the first week, the pains disappeared, and the deformity also commenced markedly and progressively to disappear. Seven months later it is noted that the deformity is all gradually disappearing, and satisfactory progress still characterises the case.

Dr. Ritchie[2] records a case of this disease of a woman aged forty, who having been treated by bone-marrow without any good result, had öophorectomy successfully performed, and after that recovery was uninterrupted. Ten days after the operation all pain had disappeared, and she was even able to turn in bed, while four months later she was able to walk two miles. Before the operation was resolved upon every possible treatment had been tried, including both white and red bone-marrow. The frequent recurrence of pregnancy and also lactation seem to favour the production of the disease.

**Deciduoma Malignum.**—Under this name it seems certain that a new disease or group of diseases has been described. Dr. John Phillips[3] says it may be defined as a malignant change, with subsequent metastasis, taking place in the remains of the products of conception, whether they be placental, molar (simple or hydatiniform), or tubal. Whether the malignant change is of a carcinomatous or sarcomatous nature, and whether arising in the fœtal or maternal portion of the placenta are at present debated points. Although the pathology is so obscure the clinical history is fairly clear. A very large number of the cases occur in young women; one has been reported in a girl of seventeen. A molar pregnancy seems more prone to malignant change than any other, uterine hæmorrhage more or less profuse occurring at a varying period after labour or abortion, is the earliest and most constant symptom; the bleeding is not continuous, but rather may be noted as coming on suddenly and without warning; fœtid discharge, with the passage of shreds, may alternate with this.

On examining the interior of the uterus, a more or less friable reddish mass can be scraped away with the finger, leaving a distinct depression in the uterine wall. Rapid metastasis soon takes place into various organs, and probably through both veins and lymphatics; these deposits are most frequently found in the lungs and the vagina. The course of these cases rapidly tends to a fatal termination, six to seven months being the extreme limit. When the diagnosis is made, treatment must be prompt, and consist in total extirpation of the uterus (if mobile) and its appendages; repeated curettings and intra-uterine applications must never be resorted to.

Apfelstedt[4] and Aschoff report two cases. Dr. J. Whitridge Williams[5] reports a case of this disease in a negress of thirty-five; she had had five confinements (the third of which was a miscarriage). The labour was slow but normal, but subsequently she was much prostrated, and her temperature remained at about 100 deg. A fortnight later a painful nodule appeared on the right labium majus, which gradually increased in size and ulcerated until it became a large sloughing mass which occupied nearly the whole of the right labium and the adjacent tissue, in the centre of which was a large fistulous opening into the rectum through which pieces were discharged. Death ensued in about three months from septicæmia. At the necropsy, in addition to the local mass, the left lung was seen to be occupied by a large number of irregularly-shaped round or oval nodules, varying in size from that of a pea to a walnut. They were greyish-red in colour; some presented soft, greyish areas in their centre. They easily broke down under the finger, were readily enucleated from the surrounding lung substance, and closely resembled placental tissue in appearance. The right lung was likewise studded with similar nodules, and the liver, spleen, and kidneys were all affected. Dr. Williams, after a most careful microscopic search, comes to the conclusion that he had to deal with a remarkable new growth. It is said that Sänger eight years ago described a similar case. Some British authorities are inclined to look upon the disease as simple sarcoma aggravated by gestation.

**Ectopic Gestation.**—Dr. A. Brothers[6] reports a case unusual in some respects. The patient was 37 years of age, had had six children, and the youngest was only sixteen months old. She then thinks herself pregnant and takes the child from the breast. Thirty-one days later hæmorrhage commences, and then continues in an irregular manner. The rule in these cases is, that generally there is a history of sterility for some years. He found that she had an indistinct but quite tender mass in the right iliac condition.

On opening the peritoneal cavity a large quantity of dark blood oozed through the incision. It was mixed with clots of various sizes. The left tube and ovary were normal. On the right side a tumour was discovered, about the middle third of the tube, about the size of a hen's egg; the finibriated extremity of the tube was adherent to the intestine, and its opening clogged with blood. This was all removed, except a portion of the ovary, which was left behind. She remained in a critical condition for three days; after that her recovery was uneventful, and she was discharged entirely cured after five weeks.

The specimen consisted of a large, dark red solid blood-clot distending the tube. No fœtus was discovered. It was probably an example of "tubal mole" or "apoplectic ovum."

Dr. Gill Wylie[7] reports a case in a girl of twenty in her first pregnancy. She came to him with continuous slight hæmorrhage, and examination led him to suspect ectopic gestation, and on operating when the abdomen was opened black clots were met with and free hæmorrhage into the peritoneal cavity. This shows that in all probability hæmorrhage begins in the early weeks of pregnancy, and therefore no treatment other than laparotomy can be anything but dangerous. He thinks also that to prevent hæmorrhage in these cases in operating one ought to proceed as in the removal of the uterus, ligating the arteries separately and making two distinct pedicles, one for the ovarian and one for the uterine artery.

Mr. Harrison Cripps[8] reports ten cases of ectopic gestation operated on with only one death out of the ten. In the fatal case the placenta had been left behind and was already beginning to decompose on the third day after the operation.

Mr. Albert Doran[9] reports three cases of the same disease all successfully operated upon, and remarks that these cases tend to show that sudden attacks of pain, associated with the development of a mass on one side of the uterus, are the most characteristic symptoms of early ectopic gestation. The signs of normal pregnancy may be absent; amenorrhea is very unreliable; nor must we forget that menstruation may continue during normal pregnancy. The breasts are an uncertain guide in this disease. The decidua is a valuable bit of evidence if it be shed, detected, and preserved. Pilliet[10] has recently shown that in early ectopic gestation the decidua is not smaller, but, on the contrary, much more developed than in normal pregnancy.

[1] Edinburgh Medical Journal, May, 1896. [2] Edinburgh Medical Journal, 1896. [3] Practitioner, April, 1896. [4] Archiv für Gynäk, 1896, Bd. 1, Hft, 3. [5] Lancet, March 28, 1896. [6] New York Medical Journal, Feb. 8, 1896. [7] New York Medical Journal, Feb. 8, 1896. [8] British Medical Journal, March 28, 1896. [9] Lancet, March 28, 1896. [10] Annales de Gynécologie et d'Obstétrique, vol. xliv., 1895, p. 241.

